# Predicting Plastid Marker Variation: Can Complete Plastid Genomes from Closely Related Species Help?

**DOI:** 10.1371/journal.pone.0082266

**Published:** 2013-11-29

**Authors:** Tiina Särkinen, Morvah George

**Affiliations:** 1 Life Sciences Department, The Natural History Museum, London, United Kingdom; 2 Royal Botanic Garden Edinburgh, Edinburgh, United Kingdom; 3 Millenium Seed Bank, Royal Botanic Gardens, Kew, United Kingdom; Swiss Federal Institute of Technology (ETH Zurich), Switzerland

## Abstract

Rapidly evolving non-coding plastid regions (NCPs) are currently widely used in evolutionary biology especially in plant systematic studies where NCPs have become one of the most commonly used tools in clarifying species relationships. Currently, the generally small amount of sequence variation provided by NCPs compared to nuclear regions makes plastid phylogeny reconstruction challenging at the species-level, especially so in species rich clades such as *Solanum* with c. 1,200 species. Previous studies have established that the set of most highly variable NCPs vary between major plant families, and here we explore whether this variation extends beyond family level to genera and major clades within genera. Using full plastome data, we identify the most highly variable plastid markers in the Potato clade of *Solanum*. We then compare sequence variation between the Potato and the closely related Morelloid clade. Results show that whilst a narrow set of NCPs show consistently high variation, levels of sequence variation in most NCPs differ greatly between the two closely related clades. The high variation detected between closely related groups implies that repeated screening studies will be needed for individual projects despite the potential availability of results from closely related taxa, and indicates a narrower applicability of family-specific screening studies than previously thought.

## Background

Rapidly evolving non-coding plastid regions (NCPs) are currently used in a wide range of evolutionary studies, including origin of domesticated species [1]–[4], diversification patterns in biodiversity hotspots [5]–[6], effect of climate change on biodiversity [7]–[8], and molecular barcoding [9]–[10]. In plant systematics, NCPs have become one of the most commonly used tools in elucidating species relationships, especially so in groups with complex evolutionary histories involving hybridisation, polyploidy and/or introgression, where plastid gene trees are used as baseline data for resolving true species trees due to their uniparental inheritance [11]–[13].

Despite past efforts to identify the most rapidly evolving plastid markers across land plants [10], [14]–[16], the generally small amount of sequence variation provided even in the most fast evolving NCPs as compared to nuclear regions is still limiting their use in phylogenetic studies. This generally low variation found in NCPs compared to more rapidly evolving nuclear regions means that more sequencing is needed to achieve equivalent resolution, making the use of plastid markers costly and time consuming especially so in large species-rich groups [17]–[18]. In fact, in many large groups, lack of plastid sequence variation has led investigators to re-direct their efforts towards the nuclear genome to look for more highly variable, single copy nuclear regions which could be used as a more cost-efficient way to derive robust phylogenies [18]–[23].

The advances in next generation sequencing technologies (NGS) during the past five years have brought solutions for building species-level plastid phylogenies. Whole plastid genomes can now be generated for multiple species with much reduced cost through massive parallel sequencing [24]–[26]. Most systematisists have yet to capitalise on NGS approaches largely due to a lack of technical expertise required to assemble and analyse NGS data. Studies are, however, now appearing, and are showing the usefulness of full plastome data in resolving broader level questions at family or order level [25], [27]–[29], in exploring sequence variation within species [30]–[31], and in elucidating relationships between closely related species [32].

Although full plastid genome sequencing has become an increasingly realistic option for molecular phylogenetic studies of relatively small genera with up to 150 species (e.g., *Pinus* [32]), such solutions are yet not practical for larger genera such as *Solanum* which includes c. 1,200 species. For such species-rich clades, cost of sequencing full plastid genomes is still large, and there are clear advantages of sequencing more species with fewer base pairs compared to fewer species with full genomes in molecular systematic projects. A trade-off approach has been adopted where only a few full plastid genomes are generated, and then used for developing highly variable plastid markers that can be sequenced with traditional Sanger sequencing. Such a screening approach has thus far been used in Solanaceae, Asteraceae, and Poaceae to identify a set of most variable plastid markers [29], [33]–[35]. Interestingly, results from these studies have revealed that there is considerable variation and surprisingly little (c. 12–25%) overlap in the most variable markers between families [29], [33]–[35]. These findings indicate that there may be only a few universally variable NCPs across land plants, and that more individual, family-specific screening studies will be required to identify the most highly variable markers for individual clades.

In this paper we explore whether variation in the most highly variable plastid markers extends beyond family level to genera and major clades within genera. We specifically ask: (1) Do genera, and clades within genera, share the same hyper-variable plastid regions, and (2) Can full genome sequences of closely related species be used to find and develop new hyper-variable plastid markers for related taxa? We use the mega-diverse genus *Solanum* as our case study, a genus in which traditionally used plastid markers have proven to provide little variation. We identify the most rapidly evolving NCPs based on the three available full plastid genomes, all from the Potato clade of *Solanum* (*S. tuberosum* L., *S. bulbocastanum* Dunal, and *S. lycopersicum* L). We then compare sequence variation in the most variable regions within the Potato clade with sequence variation in the same set of markers in the relatively closely related Morelloid clade of *Solanum*. In addition, we screen seven NCPs which have been previously used in molecular systematic studies in Solanaceae. We discuss variation across the newly identified and previously used NCPs in the context of plastid marker selection for phylogeny reconstruction.

## Results

### Full Plastome Screening

**Figure 1:**
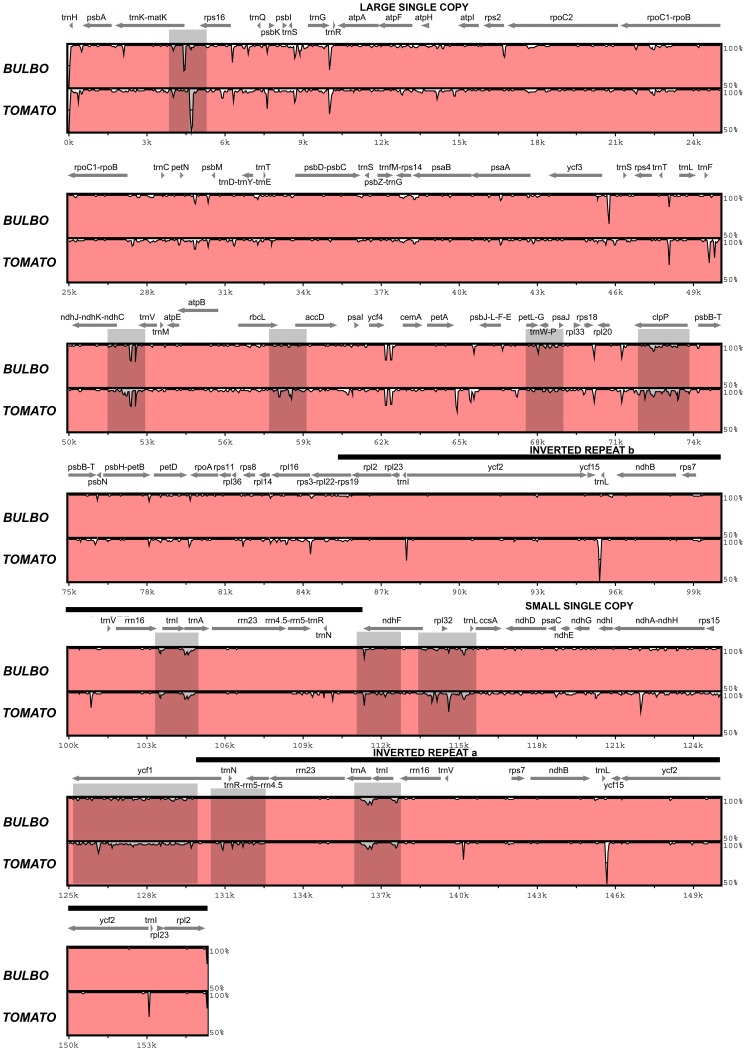
Sequence variation across the Solanum full plastome sequences. Sequence variation across the three full plastid genomes of Solanum (S. tuberosum, S. bulbocastanum, and S. lycopersicum). The graph shows sequence similarity (%, shown in pale red) in relation to the reference sequence of S. tuberosum which was used to annotate the alignment. Grey arrows above the alignment indicate genes and their orientation. Thick black lines show the position of the Inverted Repeat (IR) regions. The most variable regions detected based on a sliding window analysis are highlighted in grey. BULBO  =  S. bulbocastanum, TOMATO (S. lycopersicum).

The thirteen most variable plastid markers identified from the full plastome sequences of three Solanum species included atpB-rbcL, clpP-psbB, ndhF, ndhF-rpl32, petL-psaJ (petL-petG-trnW-trnP-psaJ), petN-psbM, rpl32-trnL, rpoC1-rpoB, trnA-trnI, trnK-rps16, and ycf1 (parts 1–3) (Table 1, Figure 1). Three of these regions included considerable proportions of coding regions: ndhF (92% coding), petL-psaJ (27% coding), and ycf1 (parts 1–3) (all coding). Most substitutions observed within these coding regions were synonymous, and Ka/Ks ratios indicated neutral or purifying selection.

**Table 1:** Screening results.

	Morelloid clade				Potato clade					
PLASTID	Total characters	Variable (%)	Variable + indels (%)	Rank	Total characters	Variable (%)	Variable + indels (%)	Rank	Shaw et al. (2005, 2007)	New regions
*ndhC- trnV*	884	**56 (6.3)**	**80 (9.0)**	1	852	12 (1.4)	17 (2.0)	14	x	
*ycf1* part 1*	1260	**64 (5.1)**	**71 (5.6)**	2	1251	**39 (3.1)**	**39 (3.1)**	3		x
*ycf1* part 2*	1293	**64 (4.9)**	**70 (5.4)**	3	1393	24 (1.7)	27 (1.9)	8		x
*rpl32-trnL*	1019	**54 (5.3)**	**62 (6.1)**	4	982	**36 (3.7)**	**44 (4.5)**	2	x	
*ycf1* part 3*	1032	**51 (4.9)**	**53 (5.1)**	5	1037	**27 (2.6)**	**28 (2.7)**	5		x
*petL-psaJ*	1217	**48 (3.9)**	**52 (4.3)**	6	1185	**23 (1.9)**	**28 (2.4)**	6		x
*trnK-rps16*	746	36 (4.8)	42 (5.6)	7	735	15 (2.0)	18 (2.4)	13	x	
*ndhF -rpl32*	772	36 (4.7)	40 (5.2)	8	773	22 (2.8)	24 (3.1)	12	x	
trnT-L*	1109	32 (2.9)	38 (3.4)	9	1117	10 (0.9)	13 (1.1)	16		
*ndhF*	967	34 (3.5)	34 (3.5)	10	980	23 (2.3)	24 (2.4)	10		
*trnS-G*	692	26 (3.8)	34 (4.9)	11	699	9 (1.3)	13 (1.9)	15	x	
*atpB-rbcL*	1266	22 (1.7)	27 (2.1)	12	1245	23 (1.8)	24 (1.9)	11		x
*clpP-psbB*	1139	21 (1.8)	27 (2.4)	13	1149	**43 (3.7)**	**48 (4.2)**	1		x
trnL-F*	549	22 (4.0)	27 (4.9)	14	544	3 (0.6)	4 (0.7)	18		
*psbK-I*	541	15 (2.8)	16 (3.0)	15	537	5 (0.9)	7 (1.3)	17		
*trnA-trnI*	1265	3 (0.2)	4 (0.3)	16	1267	**32 (2.5)**	**35 (2.8)**	4		x
*petN-psbM* ^1^	–	–	–		999	24 (2.4)	28 (2.8)	7		x
*rpoC1-rpoB* ^1^	–	–	–		980	24 (2.4)	26 (2.7)	9		x
Average		34 (3.6)	40 (4.2)			22 (2.2)	25 (2.5)			
										
**NUCLEAR**										
ITS	614	72 (11.7)	83 (13.5)		622	63 (10.1)	76 (12.2)			
*waxy* (partial)	715	67 (9.4)	82 (11.5)		704	47 (6.7)	61 (8.7)			

^1^primer design for the Morelloid clade failed for these regions.

Comparison of sequence variation in a set of highly variable plastid markers between the Potato and the Morelloid clades of *Solanum*. Top six most variable markers are shown in bold, and top three are highlighted in grey. Ranking is based on the absolute number of variable characters including indels. Larger regions marked with asterisks (*) were split into two or three parts in order to make their values comparable to other regions.

Of the thirteen markers, four (*ndhF-rpl32, rpl32-trnL,* and *trnK-*rps16, *ndhC-trnV*) were amongst the widely used plastid markers published by Shaw et al. [16], of which *trnK-*rps16 was identified as the most variable plastid marker in *Solanum* in a previous study that focused on exploring plastid variation within Solanaceae but not within *Solanum* in particular [33]. One of the markers identified, *ndhF*, has been widely used in molecular phylogenetic studies in Solanaceae [57]. The remaining regions have not been previously used in Solanaceae.

### Traditional Screening

The most variable markers identified from the full plastid genomes of the Potato clade of *Solanum* were screened in the closely related Morelloid clade together with a set of already published, commonly used plastid markers in species-level phylogenetic studies (Table 1). There was no notable length variation in any of the regions between the two clades (Table 1). Three regions, namely *ycf1* parts 1 and 3, and *rpl32-trnL*, performed consistently well and were ranked within the top six most variable regions for both clades (Table 1). The results were the same whether considering the pure number of variable sites, or variable sites including indels (Table 1). Variation was not always correlated with the length of the region: Results agree for most of the top six regions whether considering the actual number or percentage of variable sites, with the exceptions of *ycf1* part 3 and *petL-psaJ* which both show high number of actual variable sites but lower percentage values compared to *trnK-rps16* and *ndhF-rpl32* (Table 1). Because our aim was to find markers that would provide the maximum number of variable characters for phylogeny estimation with minimum sequencing cost, we focus our discussion on the top ranking regions based on the pure number of variable sites rather than the percentage.

The top three most variable plastid regions identified in the Potato clade did not correspond to the three most variable regions detected in the Morelloid clade, independent of how variation was measured (Table 1). The three most variable regions in the Potato clade included *clpP-psbB, rpl32-trnL,* and *ycf1* part 1, whilst *ndhC-trnV*, and *ycf1* parts 1 and 2 were the most variable regions in the Morelloid clade (Table 1). The most variable region in the Potato clade, *clpP-psbB*, ranked 13^th^ in the Morelloid clade (Table 1). Similarly, the most variable region in the Morelloid clade, *ndhC-trnV*, ranked 14^th^ in the Potato clade (Table 1).

### Variation within the Inverted Repeat

Some of the most highly variable markers detected in the Potato clade were found within the Inverted Repeat region (IR) of the plastid genome, including *trnA-trnI* and *ycf1* part 3. We included the counterpart of *trnA-trnI* from the IR copy B in our screening (*trnI-trnA*) in order to assure that the two repeat segments are identical, which is expected based on the fact that the two IR copies evolve in concert [36]. Both copies were found identical in reverse complement in the two clades, and hence only *trnA-trnI* results were scored (Table 1). We did not sequence both copies of the *ycf1* part 3 but assumed that these would be identical due to the concerted evolution of IR.

### Plastid versus Nuclear Sequence Variation

The average number of variable sites, including indels, in the most variable plastid markers identified in the Potato clade was 25 (2.5%), and 40 (4.2%) for the Morelloid clade (Table 1). The best performing plastid marker provided 48 (4.5%) variable sites including indels in the Potato clade, and 80 (9.0%) in the Morelloid clade (Table 1). The plastid marker variability (Potato 4.5%, Morelloid 9.0%) was still lower in both *Solanum* clades when compared to variation found in the most commonly used nuclear markers ITS and *waxy* (Potato 12.2% and 8.7%, respectively; Morelloid 13.5% and 11.5%, respectively) (Table 1). The difference between the plastid and nuclear markers was only slight, however, when comparing the actual numbers of variable characters, including indels: the most variable plastid marker provided 80 variable sites, including indels, in the Morelloid clade, whilst ITS and *waxy* provided 83 and 82 sites, respectively (Table 1).

## Discussion

A study by Daniell et al. [33] compared four plastid genomes across Solanaceae and identified 21 most variable intergenic regions within the family. Their study included two *Solanum* plastid genomes, *S. bulbocastanum* and *S. lycopersicum*, but did not specifically aim to identify the most variable plastid regions for *Solanum*. Since their study, the potato plastid genome has become available, and by comparing the three plastomes, we aimed to identify the most variable plastid regions for *Solanum* that could be used for building robust plastid phylogenies in species-level studies.

### Variation across Clades

Whether measured by variable sites, or by the combination of variable sites and indels, our results show that the set of most variable plastid regions vary between the two closely related clades of *Solanum*. In fact, most markers with high sequence variation in one clade showed more modest levels of variation in the related clade. Our results are in agreement with previous studies that have aimed to identify plastid regions that show consistently high sequence variation across a wide range of plant groups [15]–[16], [33]–[34]. These studies have acknowledged that there is no single set of NCPs that can be universally applied in species-level systematic studies across plant taxa [15]–[16]. In fact, comparison between the various family-specific screening studies showed that there are large differences between the most variable plastid regions in terms of sequence variation between major plant lineages and families [33]–[34], but the fact that large variation exists even between closely related clades within genera has not been discussed.

Although we present only a small case study, our results have implications for all studies aiming to identify a set of most variable NCPs based on only a few full plastid genomes. The differences found in the most variable plastid markers even between closely related groups, such as found here in the two *Solanum* clades, indicate that there is little universality in sequence variation in the plastid genome even at this close taxonomic level. The results imply that screening studies will be needed for individual projects despite the availability of results on plastid marker variability from a related group. For example, investigators working with other clades of *Solanum* should perform their own screening study in order to find the most variable set of plastid markers for their group of interest. Investigators working at species-level in specific clades within families for which general screening studies have been done (e.g., Asteraceae [34] and Poaceae [29], [35]) should continue to explore sequence variation at a lower taxonomic level and not draw conclusions only based on the overall patterns of sequence variation observed across these families.

### Variation within the Inverted Repeat

Unexpectedly, results from our full plastome screening showed that two IR regions, *trnA-trnI* and *ycf1* part 3, were amongst the most highly variable plastid markers in the Potato clade of *Solanum*, and in the case of *ycf1* part 3, also in the Morelloid clade. In order to assure that the two segments of the IR are evolving in concert as expected, we sequenced both IR copies of the *trnA-trnI* intron. Both copies were found to be identical, indicating that the IR structure is unchanged. What explains the elevated sequence variation within the two IR regions *trnA-trnI* and *ycf1* remains largely a mystery, because the IR is generally extremely slowly evolving amongst angiosperms [37].

In the case of the large gene *ycf1* that spans over the SSC and IR, studies have shown it to be amongst the most highly variable regions in several plant families [30], [32], [38]–[39]. The use of *ycf1* in molecular studies might be limited, however, due to evidence that it is evolving under strong positive selection in some groups [32]. No evidence was detected here that the *ycf1* is evolving under positive selection in Solanaceae based on K_a_/K_s_ ratios, but its use in molecular phylogenetic studies should be viewed with caution.

### Plastid versus Nuclear Sequence Variation

We identified ten new highly variable plastid markers based on the full plastome comparison of the three available genomes of the Potato clade of *Solanum*. These new plastid markers are now available for screening studies for molecular systematic projects on *Solanum*, and based on our preliminary tests, the new primers can also be used across Solanoideae and in some cases across the whole of Solanaceae. The new markers not only provide help in finding highly variable plastid markers needed for building robust, densely sampled species-level phylogenies, but can be used for finding potential barcodes for particular clades within the mega-diverse genus [40]. Ultimately, however, full plastome sequencing with next-generation sequencing technologies [32] will be the most cost-effective way of producing plastid sequence data, but meanwhile, highly variable short sequence reads can be expected to remain in use at least in small scale studies, as well as in studies relying on highly fragmented starting materials such as museum DNA and scatsample studies [41]–[42].

Despite our best efforts to identify highly variable plastid regions in *Solanum*, the best performing markers in both the Potato and the Morelloid clades were still marginally outperformed by the commonly used nuclear markers ITS and *waxy* in terms of the amount and percentage of sequence variation. The differences are small, however, especially so in the Morelloid clade where the best performing plastid marker contained 80 variable sites compared to ITS and *waxy*, which had 83 and 82 variable sites, respectively. Availability of such highly variable plastid markers for molecular systematic studies will be valuable, as they will help in making data generation more cost effective.

Particular markers merit special attention. One of the markers tested, *rpl32-trnL*, provided consistently high amounts of sequence variation across the two *Solanum* clades. This marker has been commonly used across angiosperms in both family- and species-level studies [15]–[16]. The marker has not yet been widely used in Solanaceae, but our results indicate that it should be included in initial screening studies. In fact, most plastid markers identified here show higher sequence variation compared to the markers that have been commonly used in molecular phylogenetic studies of Solanum (e.g., *trnT-F* and *psbK-I*). This indicates that considerable amount of time and money can be saved in molecular phylogenetic studies if initial screening studies are performed with additional markers.

### Implications for Barcoding Large Genera?

Our results have potential implications for species-level barcoding studies, especially so for studies involving large clades such as *Solanum*. Previous studies have demonstrated failure of a single or a small set of barcodes to discriminate between closely related species e.g., [40], [43]–[50]. Fazekas et al. [49] tested whether discrimination power could be increased by using a larger set of barcodes, but found that discriminating between closely related species failed in c. 30% cases even when up to seven plastid markers were used. Results presented here could partially explain the generally low success rate for barcoding such groups: if variation in plastid marker sequence variation is high across orders, families and even between closely related groups (genera, and clades within genera), a larger set of plastid markers will have to be used especially so in species-rich groups such as *Solanum*, as only a subset of markers are likely to show adequate levels of sequence variation throughout individual clades. Previous studies have identified that the failure of multiple barcodes in plants has not been due to lack of sequence variation provided by the chosen barcodes [51], but what has not been discussed is the phylogenetic or taxonomic depth at which observed parsimony informative characters occur. Poor discrimination power despite high sequence variation could indicate that the sequence variation retrieved with barcodes has not been at the appropriate phylogenetic level. We suspect this to be the case in many species rich groups based on our results presented here for *Solanum*.

## Conclusions

Our study highlights the conclusions of Shaw et al. [16] that instead of focusing on finding the most highly variable plastid marker(s), phylogenetic studies should keep screening a set of potential markers to eliminate the many slowly evolving regions from the more highly variable ones. Results from our study reveal that this applies not only to family-level studies but to studies working between closely related groups such as genera or clades within genera. Based on our case study in the mega-diverse genus *Solanum*, there is great variation in the set of most highly variable markers even between closely related groups. New primers for a set of highly variable plastid markers are now available that can be used to explore and screen plastid sequence variation in molecular phylogenetic studies of *Solanum* and Solanaceae in general.

## Methods

### Full Plastome Screening

All three currently available full plastome genomes for *Solanum*, including *S. tuberosum* (DQ231562), *S. bulbocastanum* (DQ347958), and *S. lycopersicum* (DQ347959) [33] were used to screen for the most variable regions across the plastid genome. Plastomes were aligned using ClustalW as implemented in BioEdit v7.0.9 [52]. Only minor manual adjustments were necessary due to the fact that there are no differences in gene content or gene order between the three *Solanum* species [33]. Variation across the full genomes was visualised using mVista [53] using *S. tuberosum* genome as a reference sequence. Sliding window analysis was run in the software DnaSP v5 [54] with a sliding window size 1,000 bp and 100 bp intervals in order to find the most variable c. 1,000 bp regions across the three genome alignment. Gaps were accounted for in calculating the window size. The top 20 regions with most variable sites were then further ranked based on the amount of variable characters, including indels. Indels were recorded based on the gap coding method by Simmons & Ochoterena [56]. Because the DnaSP sliding window analysis does not account for indels in variable characters, we manually recorded the number of indels present within each of the regions. Indels were accounted for because they are commonly found in NCPs and present a valuable source of information for phylogenetic analyses. Primers were designed for all new regions using the Primer3 software online (http://primer3.ut.ee), whilst published primers were used for regions already in use (Table 2). Primer design failed for *petN-psbM* and *rpoC1-rpoB* despite repeated efforts, and hence these regions were abandoned. The marker *ndhC-trnV* was chosen instead, because it showed high levels of both sequence divergence and high number of indels across the three full plastomes. Primer development and PCR optimisation for the other markers was easy, and good quality sequences were generated for the closely related Morelloid clade (12 species) to compare sequence variation across regions in the two closely allied *Solanum* clades.

**Table 2:** Primer details.

Region	Primer	Primer sequence	Reference
*atpB-rbcL*	atpB_F	ACA GGG GAC GAC CAT ACT TG	
	rbcL_R	GGA AAC CCC AGA ACC AGA AG	
*clpP-psbB*	clpP_F	GCG CAT GTA CGG TTC CTA AG	
	psbB_R	TCC TAA CCG AAT GAT GGT GA	
*ndhF*	ndhF2_F	TTC GCC AAT TTT CGC AAT A	
	ndhF2_R	TCC ACT CTC ACC TTA CAG AGA CA	
*ndhF-rpl32*	rpL32-R	CCA ATA TCC CTT YYT TTT CCA A	Shaw et al. [16]
	trnL^(UAG)^	GAA AGG TAT KAT CCA YGM ATA TT	Shaw et al. [16]
*petL-psaJ*	petG_F	TCG CAT TGA AAA ACC TCC TT	
	rpL33_R	AAT TTA GCC CCT TCA TGC TT	
*psbK-I*	psbK	TTA GCC TTT GTT TGG CAA G	Hollingsworth et al. [10]
	psbI	AGA GTT TGA GAG TAA GCA T	Hollingsworth et al. [10]
*rpl32-trnL*	trnL^(UAG)^	CTG CTT CCT AAG AGC AGC GT	Shaw et al. [16]
	rpL32-F	CAG TTC CAA AAA AAC GTA CTT C	Shaw et al. [16]
*trnK- rps16*	rpS16	AAA GTG GGT TTT TAT GAT CC	Shaw et al. [16]
	trnK^(UUU)^	TTA AAA GCC GAG TAC TCT ACC	Shaw et al. [16]
*trnA-trnI* (IRa)	trnL(GAU)_F	CTT TTC TTT TGC CGC ATT TC	
	trnA(UGC)_R	TCC TTT CTC GAC GGT GAA GT	
	trnA(UGC)_I_R	ACC ACG GCT CCT CTC TTC TC	
	trnL(GAU)_I_F	TCC CAT TTC GAT TTC GAG TC	
*trnI-trnA* (IRb)	trnGAU_F	CAC ACT TGG AGA GCG CAG TA	
	trnGAC_R	TCC TTG GGG TGA TCT CGT AG	
*trnL-F*	tabC	CGA AAT CGG TAG ACG CTA CG	Taberlet et al. [14]
	tabF	ATT TGA ACT GGT GAC ACG AG	Taberlet et al. [14]
*trnS-G*	trnG^(UUC)^*	GAA TCG AAC CCG CAT CGT TAG	Shaw et al. [16]
	trnS^(GCU)^*	AAC TCG TAC AAC GGA TTA GCA ATC	Shaw et al. [16]
*trnT-L*	tabA	CAT TAC AAA TGC GAT GCT CT	Taberlet et al. [14]
	tabD	GGG GAT AGA GGG ACT TGA AC	Taberlet et al. [14]
*ndhC- trnV*	ndhK_F	AGG CCA GAG ACA GAC CTA CG	
	atpE_R	CCT TTC GCC ATG CAT AAA CT	
	ndhK_I_F	TTA CCT CGA CCT AGC GAA GC	
	atpE_I_R	GGA ATT GCC ATC TCA AGA TTT	
*ycf1* part 1	Ycf1_F	TCA AAG GCG CAA AAC ATT TA	
	Ycf1_Ie_R	GTT GTG TTT GGA CGT GTT GG	
*ycf1* part 2	Ycf1_Ia_F	CGA AAG CGA CCT TCA TTT TT	
	Ycf1_R	TCA GTC GAA GCA GGA GAC AA	
*ycf1* part 3	trnA_F	AAT AAC ACG GGG AAT CTA GAA AA	
	trnA_R	AAA TGT TTT GCG CCT TTG AG	

Details of all the primers used in this study.

### Traditional Screening

The newly developed primers for the most variable plastid loci identified for the Potato clade were used to screen for the most variable regions in the closely related Morelloid clade of *Solanum* which belongs to the same major clade of *Solanum* [55]. Screening was done using twelve of the total c. 65 Morelloid species representing all five morphologically delimited sections (File S1).

In order to compare the variation found in the most variable regions within the Potato clade with the currently most commonly used markers in molecular systematic studies of *Solanum*, a further six markers were screened. These included the *trnT-L* and *trnL-F* intergenic spacers, which are amongst the most commonly used plastid markers in molecular systematic studies in *Solanum* [14], *psbK-I*, which has been proposed as a plant barcoding marker [10], and *trnD-T*, which is a commonly used marker in species-level phylogenetic studies across angiosperm groups [15]. Two nuclear regions were screened in order to provide a comparison between nuclear and plastid sequence variation. These included *waxy* (i.e., granule-bound starch synthase I gene, GBSSI), and the nuclear ribosomal transcribed spacer (ITS).

Total genomic DNA from silica dried leaves or herbarium material was isolated using the DNeasy Plant Mini Kit (Qiagen). All primers used are listed in Table 1. Reactions were carried out in 25 µl volume containing 2 µl of template DNA, 16 µl of buffer, 10 mg/ml of Bovine Serum Albumin, 1.5 mM of MgCl2, 0.2 mM of each dNTP, 0.2–0.5 µM of each primer, and 1 U of DNA polymerase. For a set of regions, including *rpl32-trnL*, *trnK- rps16*, *petL-psaJ*, *ndhC-trnV*, *trnA-trnI*, and *ycf1* (parts 1–2), diluted DNA (1∶10) showed higher PCR amplification success. PCR conditions were 94°C for 45 s, 30 cycles of 45 s at 94°C, 1 min at 55°C and 1 min at 72°C, followed by a final extension of 5 min at 72°C. For *trnA-trnI*, annealing temperature of 59.2°C was used. PCR products were purified using the Wizard® SV PCR Clean-Up System (Promega) and sequenced using the PCR primers following Big Dye chemistry. Consensus sequences were assembled using Sequencher (GeneCodes Corp., Ann Arbor, Michigan), and aligned using ClustalW with default settings as implemented in BioEdit v7.0.9 [52] with manual adjustments.

### Sequence Variation

We measured the number and proportion of variable sites within each region. The number of PI characters was only recorded for the Morelloid clade, due to the fact that PI characters cannot be recorded for alignments with less than four sequences. Previous studies have, however, shown that the proportion of PI and variable sites is positively correlated [29], and hence we are confident that variable characters alone provide a proxy for general variation useful for phylogenetic studies. Alignment files for the Morelloid and the Potato clade can be found in File S2 and File S3, respectively.

## Supporting Information

File S1 **Voucher details with Genbank numbers for all sequences generated in this study.**
(XLSX)

File S2 **Aligned sequence files for the Morelloid clade.**
(ZIP)

File S3 **Aligned sequence files for the Potato clade.**
(ZIP)
